# Development of Derivatives of 3, 3′-Diindolylmethane as Potent *Leishmania donovani* Bi-Subunit Topoisomerase IB Poisons

**DOI:** 10.1371/journal.pone.0028493

**Published:** 2011-12-12

**Authors:** Amit Roy, Sayan Chowdhury, Souvik Sengupta, Madhumita Mandal, Parasuraman Jaisankar, Ilda D'Annessa, Alessandro Desideri, Hemanta K. Majumder

**Affiliations:** 1 Molecular Parasitology Laboratory, Indian Institute of Chemical Biology, Kolkata, India; 2 Department of Medicinal Chemistry, Indian Institute of Chemical Biology, Kolkata, India; 3 Department of Biology, University of Rome Tor Vergata, Rome, Italy; Tulane University, United States of America

## Abstract

**Background:**

The development of 3, 3′-diindolyl methane (DIM) resistant parasite *Leishmania donovani* (LdDR50) by adaptation with increasing concentrations of the drug generates random mutations in the large and small subunits of heterodimeric DNA topoisomerase I of *Leishmania* (LdTOP1LS). Mutation of large subunit of LdTOP1LS at F270L is responsible for resistance to DIM up to 50 µM concentration.

**Methodology/Principal Findings:**

In search of compounds that inhibit the growth of the DIM resistant parasite and inhibit the catalytic activity of mutated topoisomerase I (F270L), we have prepared three derivatives of DIM namely DPDIM (2,2′-diphenyl 3,3′-diindolyl methane), DMDIM (2,2′-dimethyl 3,3′-diindolyl methane) and DMODIM (5,5′-dimethoxy 3,3′-diindolyl methane) from parent compound DIM. All the compounds inhibit the growth of DIM resistant parasites, induce DNA fragmentation and stabilize topo1-DNA cleavable complex with the wild type and mutant enzyme.

**Conclusion:**

The results suggest that the three derivatives of DIM can act as promising lead molecules for the generation of new anti-leishmanial agents.

## Introduction

DNA topoisomerases are ubiquitous enzymes that play a pivotal role in modulating the dynamic nature of DNA secondary and higher order structures and carry out vital cellular processes, e.g., replication, repair, recombination, transcription, integration and chromosomal segregation [1]–[3]. Topoisomerases are broadly classified into two types; type I and type II. Eukaryotic topoisomerase I is a type IB enzyme that catalyzes trans-esterification reaction by attaching to the 3′-end of the cleaved DNA by forming phosphotyrosine linkage. All eukaryotic type IB topoisomerases are monomeric comprising of a conserved DNA binding domain and a COOH-terminal domain. Interestingly, DNA topoisomerase I of kinetoplastid protozoan parasite *L. donovani* is distinct from other eukaryotes with respect to its biological properties and preferential sensitivity to many therapeutic agents [4], [5]. *L. donovani* topoisomerase I (LdTOP1) consist of two subunits, a large subunit (LdTOP1L) of 73 kDa and a small subunit (LdTOP1S) of 29 kDa. The two proteins are synthesized from two different genes and associate with each other through protein-protein interaction to form an active heterodimeric topoisomerase I within the parasite [4].

All the topoisomerase inhibitors are broadly divided into two classes. The Class I topoisomerase inhibitors referred to as “topoisomerase poisons” act by stabilizing the enzyme-DNA covalent complex (cleavable complex). A large number of topoisomerase inhibitors were developed which play a key role in cancer therapy. Topoisomerase II is the target of various anti-tumor agents, like amsacrine (m-AMSA), etoposide, teniposide and doxorubicin, which stabilize the “cleavable complex” between enzyme and DNA [2]. In contrast, DNA topoisomerase I inhibitors are very rare. The most widely studied and characterized inhibitor being camptothecin, a topoisomerase I poison [6]. DNA topoisomerases recently have emerged as principal therapeutic targets with a group of targeting agents having a broad spectrum of anti-parasitic activity [7]. Because of emergence of drug resistance in *Leishmania,* improved drug therapy of *Leishmania* infections is still desirable and there is a genuine need for developing therapeutic agents to combat drug resistance.

3, 3′-Diindolylmethane (DIM), a major acid condensation product of Indole-3-Carbinol (I3C) [8], is a novel immuno modulator that induces G1 arrest in breast cancer cells [9] and leads to apoptosis [10]. Recently, Gong et. al. have demonstrated that DIM is a topoisomerase IIα catalytic inhibitor and it also partially inhibits human topoisomerase I at high concentrations [11]. Our laboratory has shown that DIM is a potent *Leishmania* DNA topoisomerase 1 poison and does not stabilize topoisomerase II-mediated cleavage [8]. DIM also induces programmed cell death in *Leishmania* through inhibition of mitochondrial F_0_F_1_-ATP synthase [12]. Adaptation of the parasite with increasing concentrations of DIM generates random mutations in large and small subunits of *Leishmania* DNA topoisomerase IB (LdTOP1LS). A novel point mutation F270L in the large subunit has been found to be responsible for resistance of the parasite towards the compound [13].

In the present study, we have synthesized several derivatives of DIM and identified three derivatives with altered phenyl, methyl and methoxy moiety that inhibit the growth of the resistant parasites. The compounds also inhibit the catalytic activity of LdTOP1LS and stabilize topo1 DNA cleavable complex. The inhibition of topoisomerase I activity by phenyl derivative of DIM (DPDIM) is more than that of methyl derivative (DMDIM), where as methoxy derivative (DMODIM) causes lower inhibition. Docking studies provide a possible explanation for the higher efficacy of DPDIM. The results suggest that these derivatives are good candidates to be exploited in developing rational approaches to chemotherapy of human leishmaniasis and also can solve the prolonged DIM resistance.

## Materials and Methods

### Chemicals

**Figure 1:**
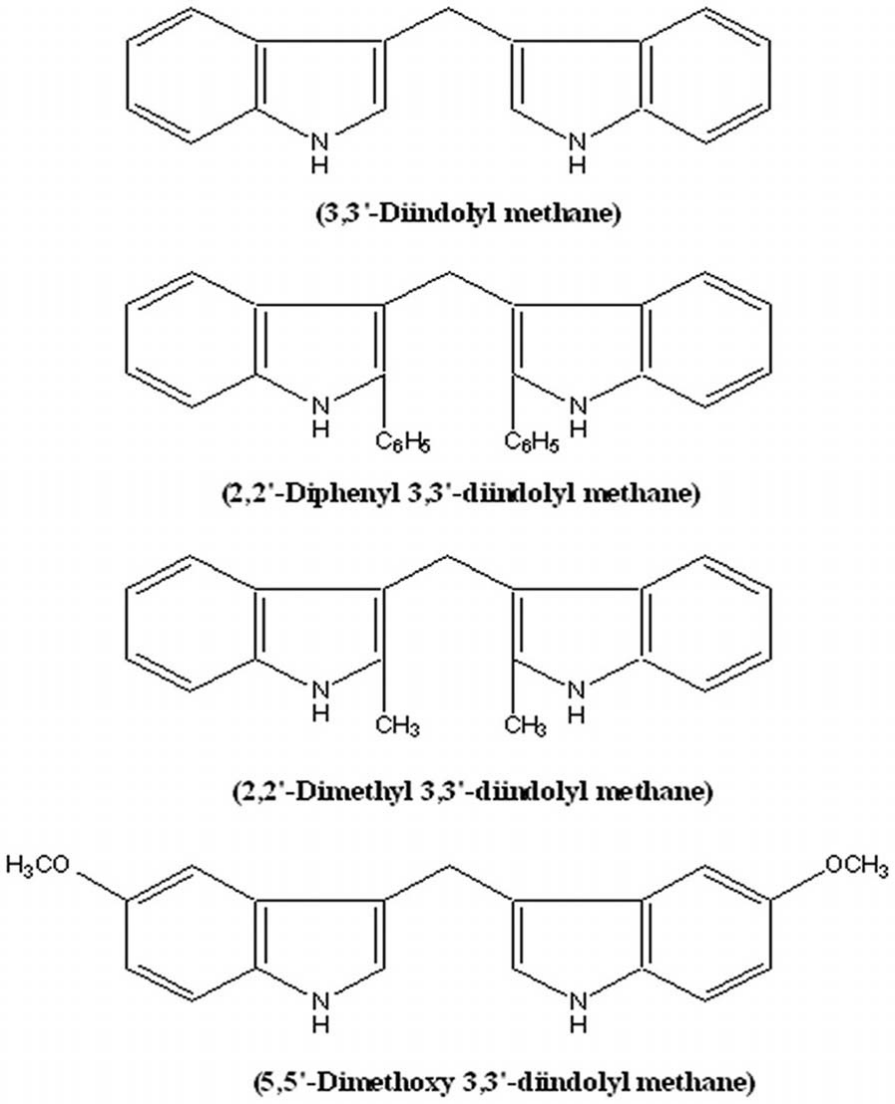
Chemical structure of DIM and its three derivatives.

The bioactive DIM (3, 3′-Diindolylmethane) and its derivatives viz. DPDIM, DMDIM and DMODIM were synthesized chemically from Indole and Urotropine as described [14]. The chemical structures of the compounds are shown in Fig. 1. All drugs were dissolved in 100% Dimethyl sulphoxide (DMSO) at 20 mM concentration and stored at −20°C.

### Cell viability assay

*L. donovani* strain AG83 and DIM resistant parasite DR50 promastigotes were grown at 22°C in M199 liquid media supplemented with 10% FBS as described [12]. Axenic amastigotes were cultured in modified M199 medium (pH 5.3) supplemented with 10 mM sodium succinate, 40 mM HEPES and 25% FCS and maintained at 37°C with 5% CO_2_.The effect of drugs on the viability of *Leishmania* parasites was determined by direct microscopic counting using light microscope (40× objective) in a haemocytometer. The cells at the exponential phase were collected and transferred into 24-well plate (3.5×10^6^ cells per well). The cells were then incubated for 12 h in the presence of various concentrations of DIM, DPDIM, DMDIM and DMODIM (1, 5, 10, 20 and 50 µM). For other experiments, 10^4^ cells were taken and grown in presence of the respective derivatives for a period of 30 days. For each 5 day interval, a small amount of aliquots were taken out. After incubation the cells were centrifuged and cell pellet was washed with PBS (1×) twice and finally suspended in 1 ml PBS (1×). Fifty µl of aliquot was taken, mixed with 0.4% trypan blue solution and incubated for 3 min at 22°C. One drop of the mixture was transferred to haemocytometer and the clear (live) and blue (dead) cells were counted. The total number of viable cells in aliquot was calculated by multiplying the total number of viable cells by 2 (the dilution factor for trypan blue). To obtain the total number of cells in the aliquot, total number of viable and nonviable cells were added up and multiplied by 2. Percentages of viable cells were calculated using the formula:
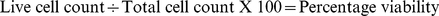



#### *In vitro* macrophage infection

Balb/c mice, originally obtained from Jackson Laboratories, Bar Harbor, ME and reared in the institute animal facilities, were used for experimental purposes with prior approval of the animal ethics committee. Macrophages were isolated from mice 36 to 48 hr post injection (intraperitoneal) with 2% (w/v) hydrolyzed starch by peritoneal lavage with ice-cold phosphate-buffered saline. Cells were washed and cultured for 18–24 hr (for adherence) in RPMI 1640 (supplemented with 100 IU/ml of penicillin and 100 mg/ml of streptomycin) containing 10% (v/v) heat-inactivated FBS (RPMI-FBS) at 37°C with 5% CO_2_ in air on sterile cover glass (22 mm×22 mm) placed in disposable plates 35 mm in diameter (Tarsons India Ltd.). The culture medium was washed off and fresh RPMI-FBS was added. About 5×10^5^ macrophages were maintained for proper distribution on cover glass. During the course of this study, macrophages were infected with promastigotes at a macrophage-to-parasite ratio of 1∶10 in RPMI-FBS for 6 hours to ensure entry of parasite. Following incubation, unphagocytosed parasites were removed by washing with medium, and cells were resuspended in RPMI-FBS at 37°C, 5% CO_2_ and then incubated for 6 hrs. Cultures were transferred to a CO_2_ incubator at 37°C and incubated for another 10–12 hr. Respective inhibitors were added at different concentrations (ranging from 5 µM to 50 µM) to infected macrophages and left for another 24 hr period. Cells were then fixed in methanol and stained with 2% Giemsa. Percentages of infected cells and total number of intracellular parasites were determined by manual counting in at least 200 cells using light microscope.

### DNA fragmentation assay for detection of apoptosis

Leishmanial cells were cultured in 24-well plates and treated with 20 µM of DIM, DPDIM, DMDIM and DMODIM for different times. Samples were collected at requisite time points and subjected to measurement of DNA fragmentations. Cytoplasmic histone-associated DNA fragments (mononucleosome and oligonucleosomes) formed during apoptosis were detected using a cell death detection ELISA kit (Roche Biochemicals) by spectrophotometric measurement of microtiter plates in a Thermo MULTISKAN EX plate reader at 405 nm. The relative percentages (with respect to samples treated with micrococcal nuclease and normalized to percentage values) were plotted as units of time or drug concentrations as described previously [12].

### Purification and reconstitution of recombinant LdTOP1LS and F270L

*E.coli* BL21 (DE3) pLysS cells harbouring pET16bLdTOP1L, pET16bLdTOP1L^F270L^ and pET16bLdTOP1S as described [8] were separately induced at O.D_600_ = 0.6 with 0.5 mM IPTG at 22°C for 12 h. Cells were lysed and proteins were purified through Ni^+2^-NTA agarose column (Qiagen) followed by phosphocellulose column chromatography (P11 cellulose, Whatman) as described [8]. Finally the purified proteins LdTOP1L, LdTOP1L^F270L^ and LdTOP1S were stored at −70°C. Purified LdTOP1L and LdTOP1L^F270L^ were mixed with purified LdTOP1S separately and at a molar ratio of 1∶1 at a total protein concentration of 0.5 mg/ml in reconstitution buffer (50 mM potassium phosphate, pH 7.5, 0.5 mM DTT, 1 mM EDTA, 0.1 mM PMSF, 10% glycerol). The mixtures were dialyzed over night at 4°C and the dialyzed fractions were used for the plasmid relaxation activity.

### Plasmid relaxation assay

The type I DNA topoisomerase was assayed by decreased mobility of the relaxed isomers of supercoiled pBluescript (SK^+^) DNA in an agarose gel. Relaxation assays were carried out with LdTOP1LS, F270LS and hTopo I in standard assay condition as described [8]. The amount of supercoiled monomer DNA band florescence after ethidium bromide (EtBr; 0.5 µg/ml) staining was quantitated by integration using Gel Doc 2000 under UV illumination (BioRad-Quality one software).

### Plasmid cleavage assay

Cleavage assay was carried out as described previously [8]. Briefly, 50 fmol of pHOT1 supercoiled DNA (containing topoisomerase I cleavage site) and 100 fmol of reconstituted LdTOP1LS were incubated in standard reaction mixture (50 µl) containing 50 mM Tris/HCl (pH 7.5), 100 mM KCl, 10 mM MgCl_2_, 0.5 mM DTT, 0.5 mM EDTA and 30 µg/ml BSA in the presence of various concentrations of drugs at 37°C for 30 min. The reactions were terminated by adding 1% SDS and 150 µg/ml proteinase K and further incubated for 1 h at 37°C. DNA samples were electrophoresed in a 1% agarose gel containing 0.5 µg/ml EtBr.

### Equilibrium cleavage assay

The 25-mer oligonucleotide 1 (5′-GAAAAAAGACTT↓AGAAAAATTTTTA-3′) was 5′-end labeled with [γ^32^P] ATP and annealed with oligonucleotide 2 (5′-TAAAAATTTTTCTAAGTCTTTTTTC-3′) to make a duplex oligonucleotide containing a topoisomerase I binding motif as described [8]. Cleavage assay was carried out using 20-fold molar excess of enzymes over duplex 25-mer DNA (enzymes, 0.2 µM; DNA, 10 nM). The reactions were carried out in standard assay condition in the absence or presence of drugs at 37°C for 30 min. All the reactions were stopped by addition of SDS to a final concentration of 2% (w/v), digested with 5 µl of 1 mg/ml trypsin and analyzed by 12% denaturing polyacrylamide gel followed by autoradiography. The amount of strand cleavage in the presence of drugs was determined by densitometry of the film as described [8].

### Immunoband depletion assay

*Leishmania* cells (3×10^7^) were cultured in absence and presence of the drugs. Nuclear fractions were isolated as described [8]. Briefly, cells were suspended in hypotonic buffer (10 mM Tris-HCl, pH 7.5, 1 mM EDTA, 0.1 mM EGTA, 1 mM PMSF, 1 mM benzamidine hydrochloride and 5 mM DTT) and homogenized. The homogenate was centrifuged for 10 min at 10,000 g. The pellets were washed and used as the source of nuclear fraction. Thereafter, the nuclear fractions were lysed with 1% SDS and subjected to SDS-PAGE (10%). The proteins were electrophoretically transferred to nitrocellulose membranes and immumobloting of immobilized proteins were carried out using a rabbit polyclonal antibody raised against LdTOP1S for topoisomerase I and ATPase domain of *L. donovani* topoisomerase II [8].

### Spectrofluorometric binding assay

Fluorescence titration was measured using Perkin Elmer, LS55, and Luminescence spectrometer. The intrinsic binding of DIM-derivatives to DNA was performed separately by fluorescence measurements at an excitation wavelength of 335 nm, 265 nm and 338 nm and emission range of 350 to 450 nm, 300 to 450 nm and 380 to 540 nm for DPDIM, DMDIM and DMODIM, respectively. Excitation and emission slit widths were 5 nm and 7.5 nm; 5 nm and 15 nm; and 5 nm and 15 nm for DPDIM, DMDIM and DMODIM, respectively. Background emission (<2%) was corrected by subtraction of spectra of blank buffer, DNA plus sample buffer and compounds plus sample buffer, respectively. Spectral titration was performed with DIM derivatives at 25°C in fluorescence buffer (20 mM Tris-HCL, pH 7.5, 50 mM NaCl and 10 mM MgCl_2_). CT DNA was added in increasing concentrations (10 to 200 µM), which are indicated in the legend. All the assays were performed in duplicate, titration points are corrected as described above and binding constants for DIM DNA-compound interaction were determined according to following equation:
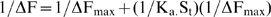
(Eq–1)where ΔF = F_x_−F_o_, F_x_ and F_o_ representing the florescence intensity of DIM derivatives in the presence or absence of the added total DNA (S_t_), respectively. ΔF_max_ is the maximum change in the fluorescence intensity. The intercept of the plot on the 1/F axis corresponding to 1/S_t_ = 0 measures the 1/F_max_, while the slope gives the estimation of the affinity constant (K_a_). The dissociation constant K_D_ = 1/K_a_.

### Molecular Docking procedure

Docking experiments have been carried out, for each DIM derivative, using as receptor the crystal structure of the LdTOP1LS complex taken from the PDB structure 2B9S [15] where residues 27–456 and 221–262 of the large and small subunits respectively are present. Residues missing in the crystal structure, 427–430 of the large subunit have been modeled with the program Swiss-PdbViewer v. 4.0.1 [16]. The GROMOS force field implemented in the program has been used to regularize the structure in order to avoid clashes. Further the program has been used to substitute the 22 DNA double strand present in the crystal structure, with the 22 DNA double strand present in the human Top1-DNA-topotecan ternary complex, coming from the PDB structure 1K4T [17], by fitting the two molecules on the backbone atoms. The substitution has been done since the presence of a cleaved site in the 22 double strand present in the 1K4T X-ray structure that contains the topotecan molecule permits to investigate the ability of the three compounds to interact with the LdTOP1LS-DNA cleavable complex. 250 docking runs for each compound have been carried out with the Autodock 4 program, using the AutodockTools suite v. 4 to prepare the structures of the ligands and the receptor [18] and making use of the Lamarckian Genetic Algorithm [19] to generate the docked complexes. The dimensions of the cubic simulative box that contains the protein-DNA complex are 48×48×48 Å^3^. Images have been obtained with the VMD visualization package [20].

## Results

### Derivatives of DIM exert cytotoxic effect on wild type and DIM resistant *L. donovani* promastigotes

**Figure 2:**
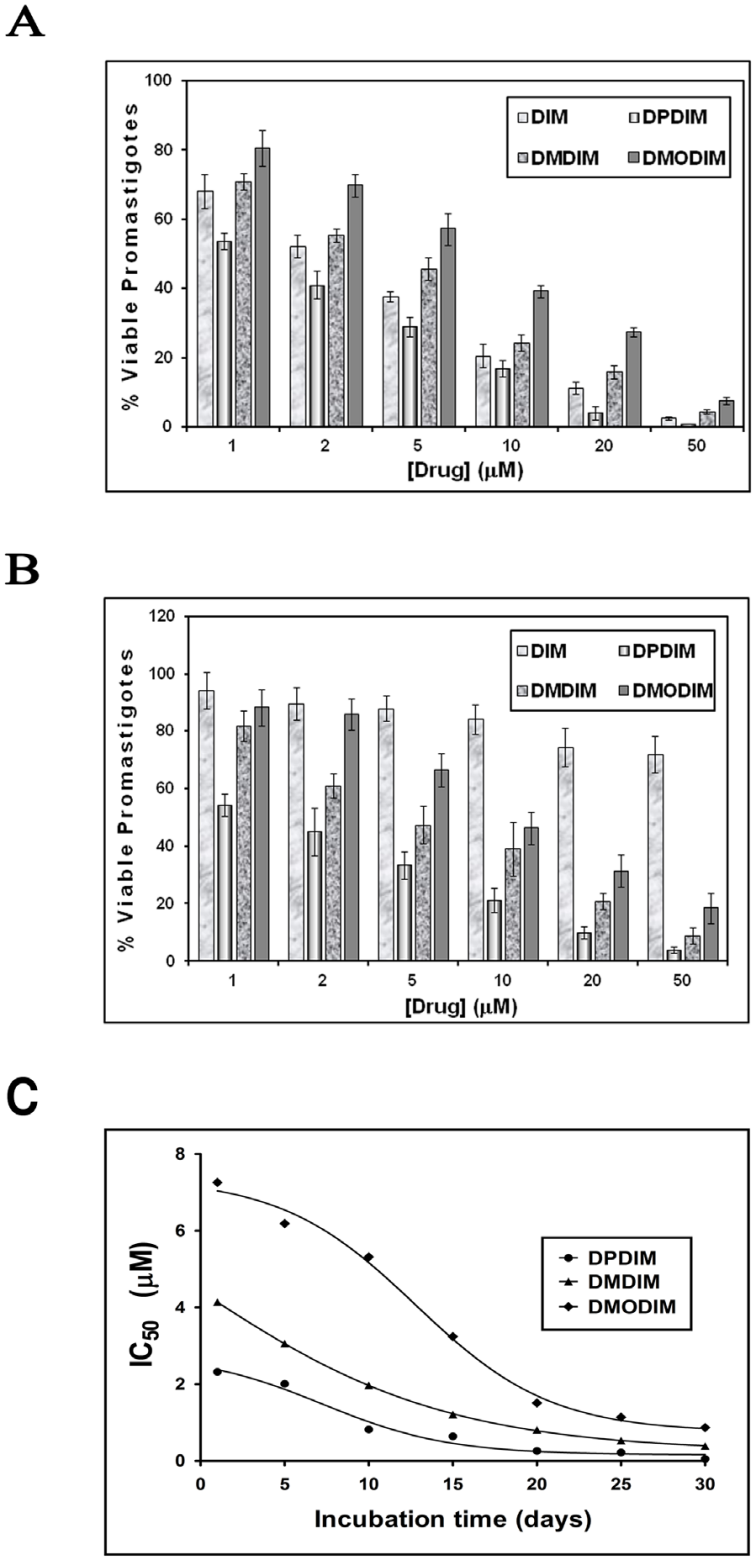
Effect of DIM-derivatives on *Leishmania* parasites. Growth of *L. donovani* AG83 promastigotes (**A**) and DIM resistant promastigotes (**B**) were monitored in presence of increasing concentrations (1, 5, 10, 20 and 50 µM) of DIM, DPDIM, DMDIM and DMODIM for 12 h. Proliferation of *Leishmania* strain in absence of treatment was considered as a maximal growth control. Change in IC_50_ values after a prolong exposure to DIM derivatives (**C**) has been assessed as described in Materials and Methods. After every 5 day a small aliquot was taken out and number of viable promastigotes was counted for each concentration. IC_50_ values have been determined from them using Graph Pad Prsim ver 5.0 software and a graphical plot was generated over time. Growth of the parasites treated with the compounds was expressed as percentage of the viable promastigotes compared with that of control. The results (percentage over control ± SD) were representative of three experiments.

The effects of different derivatives of DIM on cell proliferation were tested by incubating wild type and DIM resistant *L. donovani* promastigotes (3.5×10^6^ cells/ml) with the drugs. The resistant parasites were developed by progressive adaptation as mentioned in Materials & Methods with five different concentrations of DIM, DPDIM, DMDIM and DMODIM (1, 2, 5, 10, 20 and 50 µM) for 12 h. The number of live promastigotes was measured by differential staining of live and dead cells using light microscope under 40× objective (Fig. 2). The results indicated that the derivatives exhibited concentration dependent growth inhibitory effects on *Leishmania* parasites. At 12 h, 88%, 96% and 83% growth were inhibited by 20 µM of DIM, DPDIM and DMDIM respectively, whereas 70% growth was inhibited by 20 µM of DMODIM (Fig. 2A). In case of DIM resistant parasites 20%, 90%, 85% and 65% growth were inhibited by 20 µM DIM, DPDIM, DMDIM and DMODM respectively (Fig. 2B). The above results suggest that all the three derivatives are cytotoxic to wild type and DIM resistant *L. donovani* AG83 promastigotes.

When DIM resistant DR^50^ cells were grown continuously in presence of DIM, the parasites survived in presence of DIM and multiply in culture. But incubation with the three derivatives separately shows that the numbers of viable parasites were decreased as the incubation time is increased. DPDIM shows an IC_50_ of 2.3 µM at 24 hour incubation but after 10 days the IC_50_ value is decreased almost three times after 10 day incubation period. Further incubation up to 20 days steeply decreases the number of viable parasites and a 30 day incubation period shows an IC_50_ value of only 50 nM (Fig. 2C). So prolonged exposure of DIM derivatives (DPDIM) does not confer resistance to DIM resistant (DR^50^) promastigotes. This result is also comparable with DMDIM. After a 30 day long exposure the IC_50_ values have been changed 10 times. DMODIM has the high IC_50_ value at 24 h (7.26 µM) and has a steepest change in IC_50_ value over the 30 day exposure.

### Derivatives of DIM reduce parasite burden in *L. donovani* infected cultured macrophages and kills DIM resistant axenic amastigotes

**Figure 3:**
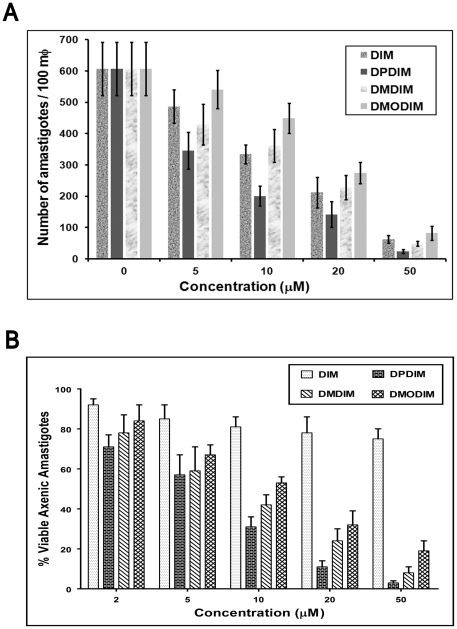
Effectiveness of clearance of internalized *L. donovani* (AG83) from *in vitro* infected mouse M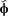. (**A**) Macrophages from peritoneal extrudate of Balb/c mouse were infected with parasites. Cultures were treated with DIM, DPDIM, DMDIM and DMODIM separately as indicated in Materials and Methods. Incubations were carried out for 24 hours. Cells were fixed, stained with Giemsa and counted under bright field microscope. Dose dependent amastigote clearance by DIM and its derivatives from infected macrophages were plotted. Experiments were carried out for three times and representative results are the mean ± S.D. (**B**) Growth of DIM resistant *L. donovani* axenic amastigotes were monitored in presence of increasing concentrations (2, 5, 10, 20 and 50 µM) of DIM, DPDIM, DMDIM and DMODIM for 12 h. Proliferation of *Leishmania* strain in absence of treatment was considered as a maximal growth control. Growth of the parasites treated with the compounds was expressed as percentage of the viable promastigotes compared with that of control. The results (percentage over control ± SD) were representative of three experiments.

Primary macrophage cells were obtained from Balb/c mice peritoneal extrudates. After adherence and inactivation, these macrophage cells were infected with early passaged *L. donovani* AG83 promastigotes were infected to macrophages. After subsequent washing they were incubated with different concentrations (5, 10, 20 and 50 µM) of each of these inhibitors for 24 h (Fig. 3A). Macrophages were fixed and intracellular amastigotes were counted by Giemsa staining. In case of DIM, DPDIM and DMDIM the efficiency of amastigote killing is upto 91%, 96% and 92% when treated with 50 µM of the drugs respectively. DMODIM is less potent in reducing the number of parasites like other three compounds. DMODIM clears about 85% of *Leishmania* parasites from macrophages.

DIM derivatives also induce death of DIM resistant axenic amatigotes. The transformation of promastigotes to axenic amastigotes was confirmed by observing under light microscopy. The effects of different derivatives of DIM on cell proliferation were tested by incubating DIM resistant *L. donovani* amastigotes (2×10^4^ cells/ml) with DIM, DPDIM, DMDIM and DMODIM separately (2, 5, 10, 20 and 50 µM) for 12 h. The number of live amastigotes was measured by differential staining of live and dead cells using light microscope under 40× objective (Fig. 3B). The results indicated that the derivatives exhibited concentration dependent growth inhibitory effects on *Leishmania* parasites. At 12 h, 98%, 92% and 79% growth were inhibited by 50 µM of DPDIM, DMDIM and DMODIM respectively (Fig. 3B).

### The derivatives of DIM induce fragmentation of genomic DNA in wild type and DIM resistant *Leishmania* parasites

**Figure 4:**
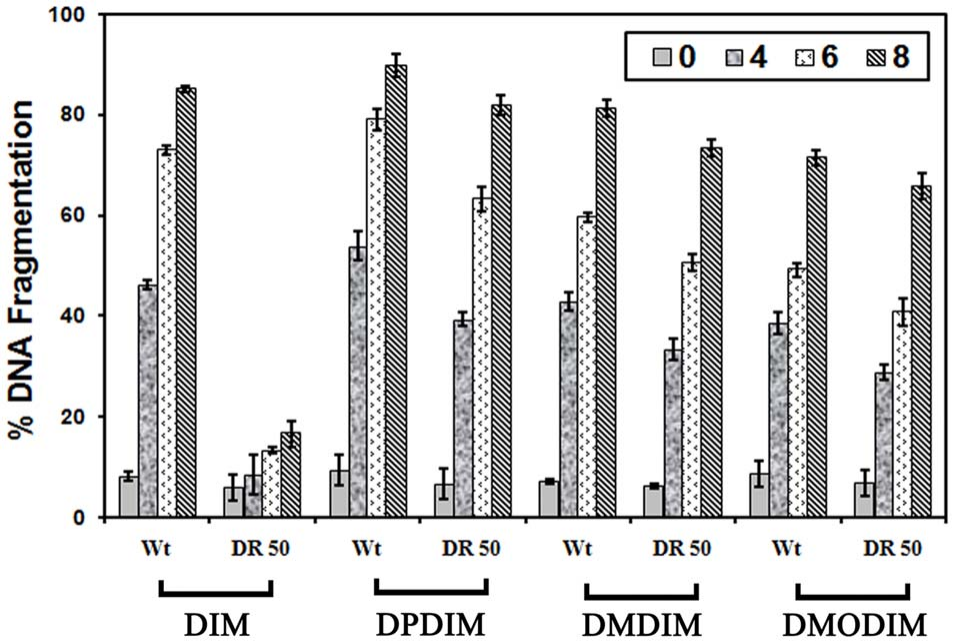
DIM derivatives-induced fragmentation of genomic DNA in wild type and DIM resistant parasites. Relative percentage of DNA fragmentation measured by cell death detection ELISA kit in L. donovani promastigotes treated with DIM, DPDIM, DMDIM and DMODIM. The experiments were performed three times and representative data from one of these experiments as means ± SD.

The internucleosomal DNA digestion by an endogenous nuclease (genomic DNA fragmentation) is considered as a hallmark of apoptotic cell death [21]. Previously, we found that DIM induces genomic DNA fragmentation in *Leishmania* parasites [12]. To investigate whether these derivatives also do the same, we performed the DNA fragmentation assay with wild type *L. donovani* AG83 promastigotes and DIM resistant parasites (Fig. 4) by ELISA as described in Materials and Methods. Results show that there were 73%, 79%, 60% and 49% fragmentation of DNA in wild type promastigotes at 6 h by treatment with 20 µM of DIM, DPDIM, DMDIM and DMODIM respectively. DNA fragmentation increased to 85%, 89%, 81% and 71% at 8 h of treatment in wild type parasites. The DIM resistant parasites (LdDR50) have much less effect in DNA fragmentation (13% at 6 h and 17% at 8 h) when treated with 20 µM DIM. On the other hand the extents of DNA fragmentations were 63%, 51% and 40% at 6 h treatment with DPDIM, DMDIM and DMODIM respectively. The extent of fragmentation increased to 82%, 74% and 65% after treatment for 8 h with the drugs as mentioned above. The above results suggest that DNA fragmentation may be responsible for drug induced cell death.

### DIM-derivatives stabilize topo I-DNA cleavable complexes formation *in vitro*

**Figure 5:**
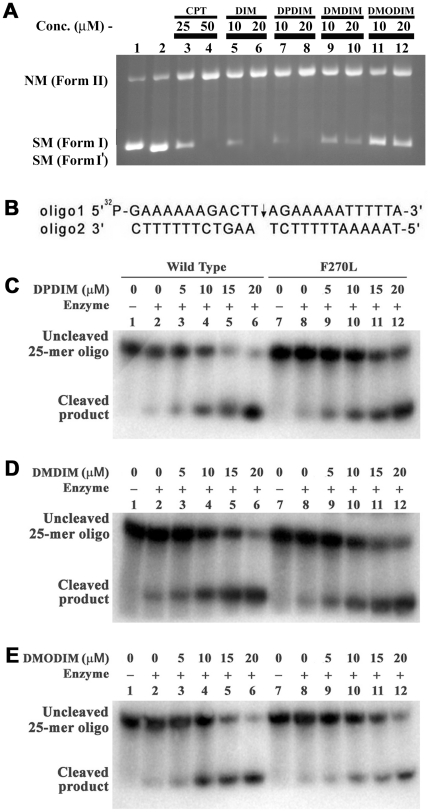
DIM-derivatives stabilize LdTOP1LS-mediated DNA cleavage. (**A**) Plasmid Cleavage experiment. Cleavage reaction and agarose gel electrophoresis were performed as described in ‘Materials and Methods’. Lane 1, 50 fmol of pHOT1 DNA; lane 2, with 100 fmol LdTOP1LS; lanes 3 and 4, same as lane 2, but in the presence of 25 and 50 µM CPT respectively as control; lanes 5 and 6, same as lane 2, but in the presence of 10 and 20 µM DIM respectively; lanes 7 and 8, same as lane 2, but in the presence of 10 and 20 µM DPDIM respectively; lanes 9 and 10, same as lane 2, but in the presence of 10 and 20 µM DMDIM respectively; lanes 11 and 12, same as lane 2, but in the presence of 10 and 20 µM DMODIM respectively. Positions of supercoiled monomer (SM; form I) and nicked monomer (NM; form II) are indicated. (**B**) Oligo sequence used for the Equilibrium cleavage assay. An arrow indicates the cleavage site. (**C**), (**D**) **and** (**E**) Duplex oligonucleotide cleavage. The cleavage reactions and electrophoresis in a denaturating polyacrylamide gel were performed as described in Materials and Methods. (**C**) Lane 1, 10 nM of 5′-^32^P-end labeled 25 mer duplex oligonucleotides as indicated above. Lane 2, same as lane 1, but incubated with 0.2 µM of wild type enzyme. Lanes 3 to 6, same as lane 2, but incubated with 5, 10, 15 and 20 µM of DPDIM respectively for 60 min at 23°C. Lane 7, same as lane 1; lanes 8, same as lane 7, but incubated with 0.2 µM of F270L mutant enzyme. Lanes 9–12, same as lane 8, but incubated with 5, 10, 15 and 20 µM of DPDIM respectively for 60 min at 23°C. (**D and E**) Same as (**C**), but incubated with 5, 10, 15 and 20 µM of DMDIM and DMODIM respectively. All the reactions were stopped by addition of SDS to the final concentration of 2% (w/v). Samples were precipitated with ethanol, digested with trypsin and analyzed by 12% denaturing polyacrylamide gel as described in Materials and Methods.

The effect of DIM and its derivatives on *L. donovani* topoisomerase I was examined by plasmid DNA cleavage assay as described [8]. The topoisomerase reaction has three general mechanistic steps: (i) binding of enzyme to the substrate DNA, (ii) single strand breakage and subsequent strand rotation through the break leading to change in linking number, and (iii) strand religation. The second step of reaction is the introduction of a single-stranded nick in the phosphodiester bond of the DNA, through which an intact strand is allowed to pass. During this process, a covalent bond is formed between the 3′-phosphoryl group of the DNA backbone and the tyrosine residue at the active site of topoisomerase I. Topoisomerase inhibition can be achieved by prevention of enzyme-DNA binary complex formation or by stabilization of enzyme-DNA cleavable complex. To investigate the ability of DIM-derivatives to stabilize the cleavable complex formation between enzyme and oligonucleotide containing a topoisomerase IB specific binding motif we carried out plasmid cleavage assay as described previously [8]. Cleavage assay was performed with LdTOP1LS and pHOT1 DNA containing a topoisomerase IB specific binding motif as described in ‘Materials and Methods’. As shown in Fig. 5A, in presence of 50 µM CPT, the closed circular DNA (form I) was totally converted to nicked circular DNA (form II, lane 4). The result was same with 20 µM DIM (lane 6), 10 µM DPDIM (lane 7) and 20 µM DMDIM (lane 10). However, treatment with 20 µM DMODIM didn't cause conversion of all the form I DNA to form II (lane 12). The above observation was further supported by duplex oligonucleotide cleavage assay as described [8]. The experiment was performed by incubating duplex oligonucleotide with topoisomerase I in presence of 5, 10, 15 and 20 µM of DPDIM, DMDIM and DMODIM. In the absence of drugs, the cleavage-religation equilibrium is shifted towards religation for the wild type enzyme (LdTOP1LS), as little trapped cleavable complex was found and most of the cleaved product is religated and migrated as uncleaved 25-mer (panels C, D and E, lane 2). When the wild type enzyme is exposed to increasing concentration of DPDIM the cleavage-religation equilibrium is shifted towards cleavage. The band corresponding to the 12-mer cleaved products is clearly detectable and the band intensity is increased with increasing concentration of DPDIM (panel C, lanes 3–6). We have previously found that the F270L topoisomerase I mutant is insensitive to DIM [13]. Therefore, we have investigated whether the three DIM derivatives could interact with the DIM-resistant F270L mutant stabilizing the topoisomerase I-DNA cleavable complex. In the presence of increasing concentrations of three DIM-derivatives the equilibrium is shifted towards cleavage as revealed by the appearance of 12-mer cleaved product on the gel for both wild type (Fig. 5C, D and E; lanes 3–6) and F270L mutant enzyme (Fig. 5C, D and E; lanes 8–12). Moreover, all the three derivatives enhanced cleavage with increasing concentrations of the drugs. DPDIM-induced cleavage increased to 67% in presence of wild type enzyme (Fig. 5C, lane 6) and 56% in presence of F270L mutant (lane 12) at 20 µM concentration with respect to the extent of cleavable complex formed without the drug in presence of wild type enzyme. At 20 µM DMDIM and DMODIM, the extent of cleavage reached approximately 61% and 56% respectively in presence of wild type enzyme (Fig. 5D and E, lane 6), and 53% and 50% in presence of F270L mutant respectively (Fig. 5D and E, lane 12). These results indicate that the three derivatives are potent topoisomerase I poisons. Moreover, although F270L is insensitive to DIM [13] and all three DIM-derivatives stabilize the cleavable complexes in F270L mutant enzyme, the binding site of DIM might not be overlapping and may be different from the derivatives.

### DIM-derivatives stabilize *in vivo* cleavable complex formation in *L. donovani* promastigotes

**Figure 6:**
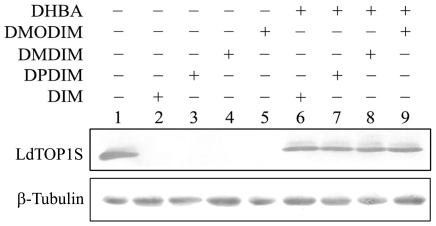
DIM-derivatives induce *in vivo* formation of topoisomerase-DNA cleavable complex within the cells. Stabilization of topoisomerase-I mediated cleavable complex was determined by immunoband depletion assay. Top panel: Immunoband depletion of *L. donovani* topoisomerase I, using an antibody raised against LdTOP1LS. *Leishmanial* cells were treated with 0.2% DMSO alone (lane 1), 20 µM each of DIM (lane 2), DPDIM (lane 3), DMDIM (lane 4) and DMODIM (lane 5) respectively. Lanes 6–9, preincubation with 20 µM DHBA before treatment with DIM, DPDIM, DMDIM and DMODIM respectively. Bottom panel: β-tubulin loading control.

We have reported earlier that DIM induces topoisomerase I and not topoisomerase II-mediated cleavable complex formation in *L. donovani* [8]. To investigate the ability of DIM derivatives to induce *in vivo* covalent complex formation between topoisomerase I and DNA in the *Leishmania* parasites we performed the immunoband depletion experiments with *L. donovani* promastigotes. Nuclear fractions were prepared from untreated as well as drug-treated promastigotes and subjected to SDS-PAGE. If topoisomerase I can form a covalent complex with genomic DNA inside the cells, then topoisomerase I-DNA cleavable complex cannot enter into the gel. On the other hand, if topoisomerase I does not form a complex with DNA and remains free, it will enter into the gel. The presence of topoisomerase I was detected by immunoblotting as described in ‘Materials and Methods’. The immunoband depletion data are summarized in Figure 6. It was observed that the immunoband of topoisomerase I disappeared in presence of 20 µM of DIM, DPDIM, DMDIM and DMODIM for 6 h incubation respectively (lanes 2–5). Preincubation with 20 µM DHBA before treatment with drugs causes reappearance of immunoband of *L. donovani* topoisomerase I as cleavable complex formation is prevented (lanes 6–9). The above results suggest that DIM is responsible for the stabilization of topoisomerase I-DNA cleavable complex inside the cells. It should be mentioned here that topoisomerase I of *Leishmania* is a heterodimer and the catalytic site (SKXXY) is present in the small subunit of enzyme (LdTOP1S), which is involved in the formation of topoisomerase I-DNA covalent complex. So we have used the antibody raised against the small subunit of *L. donovani* topoisomerase I to study the immunoband depletion assay.

To rule out the topoisomerase II mediated cleavable complex stabilization by the derivatives, we have carried out immunoband depletion assay with antibody raised against ATPase domain (43 kDa) of *L. donovani* topoisomerase II. The results (Figure S1) clearly indicate that DIM and its derivatives do not stabilize cleavage complex formation mediated by *Leishmania* topoisomerase II.

### Interaction of derivatives of DIM with DNA as studied by fluorescence quenching

**Figure 7:**
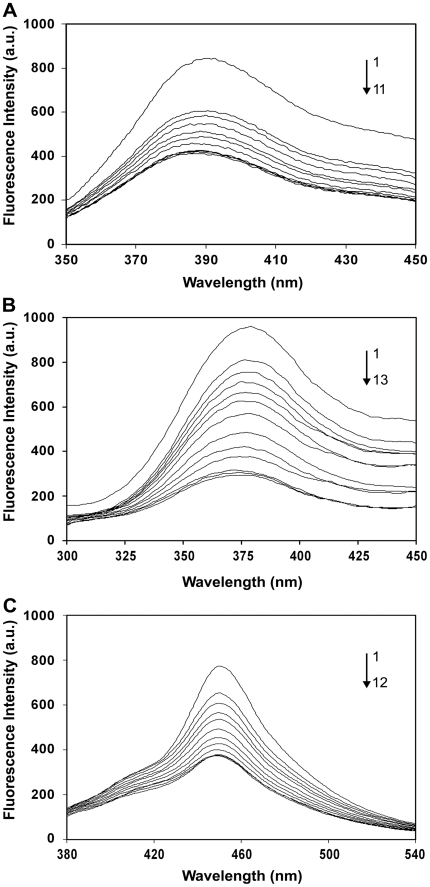
Fluorescence quenching study of DIM derivatives-DNA interaction. (**A**) CT DNA concentration-dependently quenched DPDIM fluorescence at 390 nm. (**B**) CT DNA concentration-dependently quenched DMDIM fluorescence at 379 nm. (**C**) CT DNA concentration-dependently quenched DMODIM fluorescence at 450 nm. The arrow of each figure indicates the change in the emission spectra of DPDIM, DMDIM and DMODIM respectively on addition of CT DNA.

DIM interacts with DNA with the dissociation constant (K_D_) of 2.2×10^−5^ M [8]. Therefore, it was of interest to determine whether DIM derivatives could interact with DNA. Fluorescence quenching assay was performed to determine the interaction between the derivatives of DIM and DNA at different concentrations of DNA (10 to 200 µM). DPDIM has fluorescence emission maximum (λ_em_^max^) at the wavelength of 390 nm with a λ_ex_ of 335 nm, whereas DMDIM has fluorescence emission maximum at a wavelength of 379 nm with a λ_ex_ of 265 nm and DMODIM has fluorescence emission maximum at a wavelength of 450 nm with a λ_ex_ of 338 nm. The excitation and emission slit widths were set at 5 nm and 7.5 nm for DPDIM, 5 nm and 15 nm for DMDIM; and 5 nm and 15 nm for DMODIM. Appropriate blanks corresponding to the buffer were subtracted to correct the background fluorescence. The fluorescence intensity decreases by increasing the concentration of DNA due to quenching reaction. Addition of DNA causes a slight shift of the maximum peak (λ shift) from 390 nm to 386 nm for DPDIM and from 379 nm to 374 nm for DMDIM with a concomitant decrease in fluorescence intensity (Fig. 7A and B). There is no λ shift for DMODIM (Fig. 7C). A progressive change in the fluorescence spectra of three DIM-derivatives on addition of different concentrations of DNA indicated an association between them. The dissociation constant (K_D_) calculated using Scatchard analysis [22] was 1.1×10^−5^ M for DPDIM, 6.3×10^−5^ M for DMDIM and 4.8×10^−5^ M for DMODIM. The above results indicate that the affinity of DPDIM to DNA is 2-fold greater compared to the DIM mother compound [8] and the affinity of DMDIM and DMODIM to DNA are approximately 3-fold and 2-fold less compared to DIM, respectively.

### Docking of DMDIM, DMODIM and DPDIM on the protein-DNA cleavable complex

**Figure 8:**
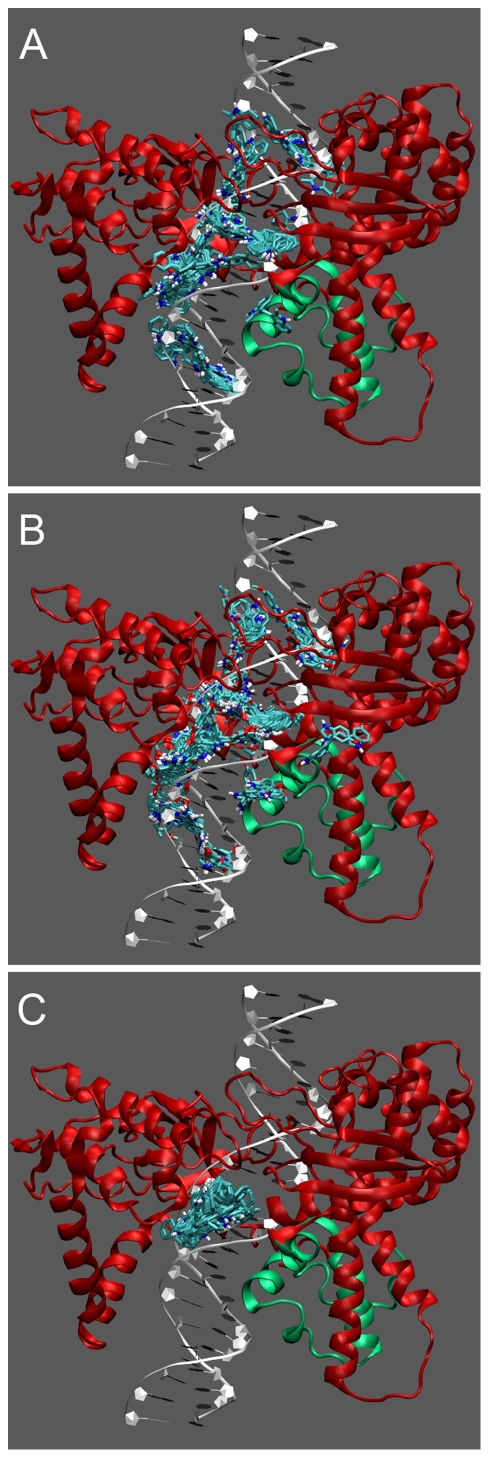
Docking results. Spread of the 250 DMDIM (A), DMODIM (B) and DPDIM (C) molecules docked on the heterodimeric LdTOP1LS-DNA complex. The protein is represented in ribbons, with the large subunit colored in red and the small subunit colored in green. The DIM derivatives are represented in licorice with the C, O and H atoms colored cyan, red and white respectively.

It is shown earlier that in the presence of these derivatives, the cleavage-religation equilibrium is shifted towards cleavage. The ability of DIM derivatives to impair the religation reaction of LdTOP1LS has been investigated through Molecular Docking, to define the interaction mode between the drugs and the protein-DNA covalent complex, as recently done to predict the inhibition mode of Erybraedin C on human topoisomerase IB [23]. Figure 8 shows the spread of the 250 docked structures for the three compounds.

DMDIM and DMODIM (Fig. 8A and B) show a similar interaction profile, in fact in both cases the docked molecule is spread out along all the DNA structure, between base pairs 5–19. All the molecules have a comparable interaction energy ranging between −6 and −10 Kcal/mol indicating that the two molecules do not have a preferential binding site and only some of them docks in proximity of the active site. On the other hand DPDIM docks in a unique binding region (Fig. 8C), in fact in all 250 runs the molecule is located in a single site. In detail, all the docked molecules are found in the DNA minor groove and the free energy of the complexes are also characterized by a very narrow range between −8.1 and −9.8 Kcal/mol. The drug binding site is localized between base pairs 10–14, relatively close to the active site, and its inhibitory effect on the religation reaction, could be explained either through an impairing of the process of strand rotation or to a direct slowing of the religation process.

## Discussion

Emergence of resistance against conventional drugs for leishmaniasis poses great problem in the treatment and therefore genuinely demands development of new therapeutics. DIM has been shown to have several pharmacological properties [8]. Our laboratory has shown that this phytochemical is a potent *Leishmania* topoisomerase I poison [8]. Therefore in order to study the mechanism of resistance and to circumvent the problem of resistance, we have developed DIM resistant *L. donovani* parasites by progressive adaptation to the drug [13] and synthesized several derivatives of DIM.

In the previous study, we have identified that DIM is a potent inhibitor of LdTOP1LS and also kills the parasites via induction of apoptosis by abrogating Fo-F1 ATP synthase function. Development of resistance to DIM is due to mutation in LdTOP1LS (F270L) and also due to overcoming Fo-F1 ATP synthase inhibition. Development of DIM derivatives as antileishmanial drugs emphasizes on the selective inhibition of DIM resistant parasites. Therefore to develop resistance against one of the DIM derivatives it is necessary to acquire gene mutation in LdTOP1LS (F270L) as well as in Fo-F1 ATP synthase. Docking experiments suggested a possible binding site of the three derivatives. In detail, DPDIM displays a higher specificity for binding than the other two compounds. It interacts with the cleavable complex at the level of the DNA minor groove. Finally it inhibits further religation step by impairing DNA intact strand rotation. This study is markedly different with that of DIM-LdTOP1LS binding. The binding causes the three derivatives to stabilize the topoisomerase I-DNA cleavable complexes *in vitro* with the wild type and F270L mutant enzyme (Fig. 5). *In vivo* cleavable complex stabilization also substantiate with the *in vitro* observations. Therefore it might be concluded from the catalytic inhibition of wild type enzyme by the three derivatives and the topo I- DNA cleavable complex formation between the mutant enzyme and the three derivatives that the binding site of DIM to enzyme is not overlapping with the derivatives.

Previously we have shown that DIM resistant parasites inherit the F270L mutation and no known drug transporters were associated with the resistance phenotypes [13]. This implies that DIM stays well within the parasites and progressive adaptation is selected by constant drug pressure. Real time PCR analysis showed that in DIM resistant parasites the levels of large and small subunits of LdTOP1LS were decreased. So there is no possibility to tie up the inhibitor by the enzyme to prevent its action. By adaption to DIM derivatives the DIM resistant parasites need to accumulate more mutation to become resistant to these derivatives. Some simple substitutions to DIM confer the DIM resistant parasites to become sensitive to these derivatives. Fluorescence quenching experiments revealed that DPDIM has 2-fold higher affinity to DNA than DIM. However, DMDIM and DMODIM have 3-fold and 2-fold lower affinity to DNA than DIM respectively. Again a long term exposure of these derivatives to DR50 cells shows that IC_50_ values were decreased as incubation time is increased. When DIM is applied to DIM resistant parasites, the number of viable parasites increases with time over a period of 30 days. But when DIM resistant parasites were treated with the derivatives, the number of viable parasites decreased with time. This experiment proves that resistance is not acquired in DR50 parasites upon prolong incubation of these derivatives. DPDIM being the strongest drug shows a loweset IC50 value and change in IC50 values over 30 days is lowest amongst the three derivatives. DPDIM kills almost all DR50 cells in 30 days incubation period supporting that development of further resistance is low if DPDIM is used for treatment.

In conclusion, the present study indicates that among all the compounds, DPDIM is the most potent antileishmanial agent and all the three derivatives are potent *Leishmania* topoisomerase I poison. Structure-function analysis of enzyme-drug interactions along with modeling studies might be exploited in developing rational approaches to chemotherapy of human leishmaniasis and can also facilitate the study of future DIM resistance.

## Supporting Information

Figure S1 **Stabilization of topoisomerase-II mediated cleavable complex was determined by immunoband depletion assay.** Immunoband depletion of *L. donovani* topoisomerase II, using an antibody raised against LdTOP2 (ATPase domain, 43 kDa). *Leishmanial* cells were treated with 0.2% DMSO alone (lane 1), 20 µM CPT (lane 2); 20 µM and 50 µM of Etoposide (lanes 3 and 4 respectively) and 50 µM of DIM (lane 5), DPDIM (lane 6), DMDIM (lane 7) and DMODIM (lane 8) respectively.
(TIF)

## References

[1] Champoux JJ (2001). DNA topoisomerases: structure, function, and mechanism.. Annu Rev Biochem.

[2] Liu LF (1989). DNA topoisomerase poisons as antitumor drugs.. Annu Rev Biochem.

[3] Wang JC (1996). DNA topoisomerases.. Annu Rev Biochem.

[4] Das BB, Sen N, Ganguly A, Majumder HK (2004). Reconstitution and functional characterization of the unusual bisubunit type I DNA Topoisomerase from *Leishmania donovani*.. FEBS Letts.

[5] Das BB, Sen N, Roy A, Dasgupta SB, Ganguly A (2006). Differential induction of *Leishmania donovani* bi-subunit topoisomerase I-DNA cleavage complex by selected flavones and camptothecin: activity of flavones against camptothecin-resistant topoisomerase I.. Nucleic Acids Res.

[6] Hsiang YH, Hertzberg R, Hecht S, Liu LF (1985). Camptothecin induces protein-linked DNA breaks via mammalian DNA topoisomerase I.. J Biol Chem.

[7] Das A, Sengupta T, Dasgupta A, Majumder HK (2004). Toppisomerases of kinetoplastid parasites as potential chemotherapeutic targets.. Trends in Parasitol.

[8] Roy A, Das BB, Ganguly A, Dasgupta SB, Khalkho NV (2008). An insight into the mechanism of inhibition of unusual bi-subunit topoisomerase I from *Leishmania donovani* by 3,3′-di-indolylmethane, a novel DNA topoisomerase I poison with a strong binding affinity to the enzyme.. Biochem J.

[9] Hong C, Kim HA, Firestone GL, Bjeldanes LF (2002). 3,3′-Diindolylmethane (DIM) induces a G (1) cell cycle arrest in human breast cancer cells that is accompanied by Sp1-mediated activation of p21 (WAF1/CIP1) expression.. Carcinogenesis.

[10] Hong C, Firestone GL, Bjeldanes LF (2002). Bcl-2 family-mediated apoptotic effects of 3,3′-diindolylmethane (DIM) in human breast cancer cells.. Biochem Pharmacol.

[11] Gong Y, Firestone GL, Bjeldanes LF (2006). 3,3′-diindolylmethane is a novel topoisomerase II alpha catalytic inhibitor that induces S-phase retardation and mitotic delay in human hepatoma HepG2 cells.. Mol Pharmacol.

[12] Roy A, Ganguly A, BoseDasgupta S, Das BB, Pal C (2008). Mitochondria dependent ROS-mediated programmed cell death (PCD) induced by 3, 3′-Diindolylmethane (DIM) through Inhibition of F0F1-ATP synthase in unicellular protozoan parasite *Leishmania donovani*.. Mol Pharmacol.

[13] Roy A, BoseDasgupta S, Ganguly A, Jaisankar P, Majumder HK (2009). Topoisomerase I gene mutations at F270 in the large subunit and N184 in the small subunit contribute to the resistance mechanism of the unicellular parasite *Leishmania donovani* towards 3,3′-diindolylmethane.. Antimicrob Agents Chemother.

[14] Pal C, Dey S, Mahato SK, Vinayagam J, Pradhan PK (2007). Eco-friendly synthesis and study of new plant growth promoters: 3,3′-Diindolylmethane and its derivatives.. Bioorg Med Chem Lett.

[15] Davies DR, Mushtaq A, Interthal H, Champoux JJ, Hol WG (2006). The structure of the transition state of the heterodimeric topoisomerase I of Leishmania donovani as a vanadate complex with nicked DNA.. J Mol Biol.

[16] Guex N, Diemand A, Peitsch MC (1999). Protein modelling for all.. TiBS.

[17] Staker BL, Hjerrild K, Feese MD, Behnke CA, Burgin AB (2002). The mechanism of topoisomerase I poisoning by a camptothecin analog.. Proc Natl Acad Sci U S A.

[18] Morris GM, Huey R, Lindstrom W, Sanner MF, Belew RK (2009). AutoDock4 and AutoDockTools4: Automated docking with selective receptor flexibility.. J Comput Chem.

[19] Morris GM, Goodsell DS, Halliday RS, Huey R, Hart WE (1998). Automated docking using a Lamarckian genetic algorithm and an empirical binding free energy function.. J Comput Chem.

[20] Humphrey W, Dalke A, Schulten K (1996). VMD: visual molecular dynamics.. J Mol Graph.

[21] Compton MM (1992). A biochemical hallmark of apoptosis: internucleosomal degradation of the genome.. Cancer Metastasis Rev.

[22] Guharay J, Sengupta B, Sengupta PK (2001). Protein-flavonol interaction: fluorescence spectroscopic study.. Proteins.

[23] Tesauro C, Fiorani P, D'Annessa I, Chillemi G, Turchi G (2010). Erybraedin C, a natural compound from the plant Bituminaria bituminosa, inhibits both the cleavage and religation activities of human topoisomerase I.. Biochem J.

