# Supplementary figures and images for: Development of Derivatives of 3, 3′-Diindolylmethane as Potent Leishmania donovani Bi-Subunit Topoisomerase IB Poisons
Source: PLoS One. 2011 Dec 12;6(12):e28493. doi: 10.1371/journal.pone.0028493 (PMC3236199; doi:10.1371/journal.pone.0028493)

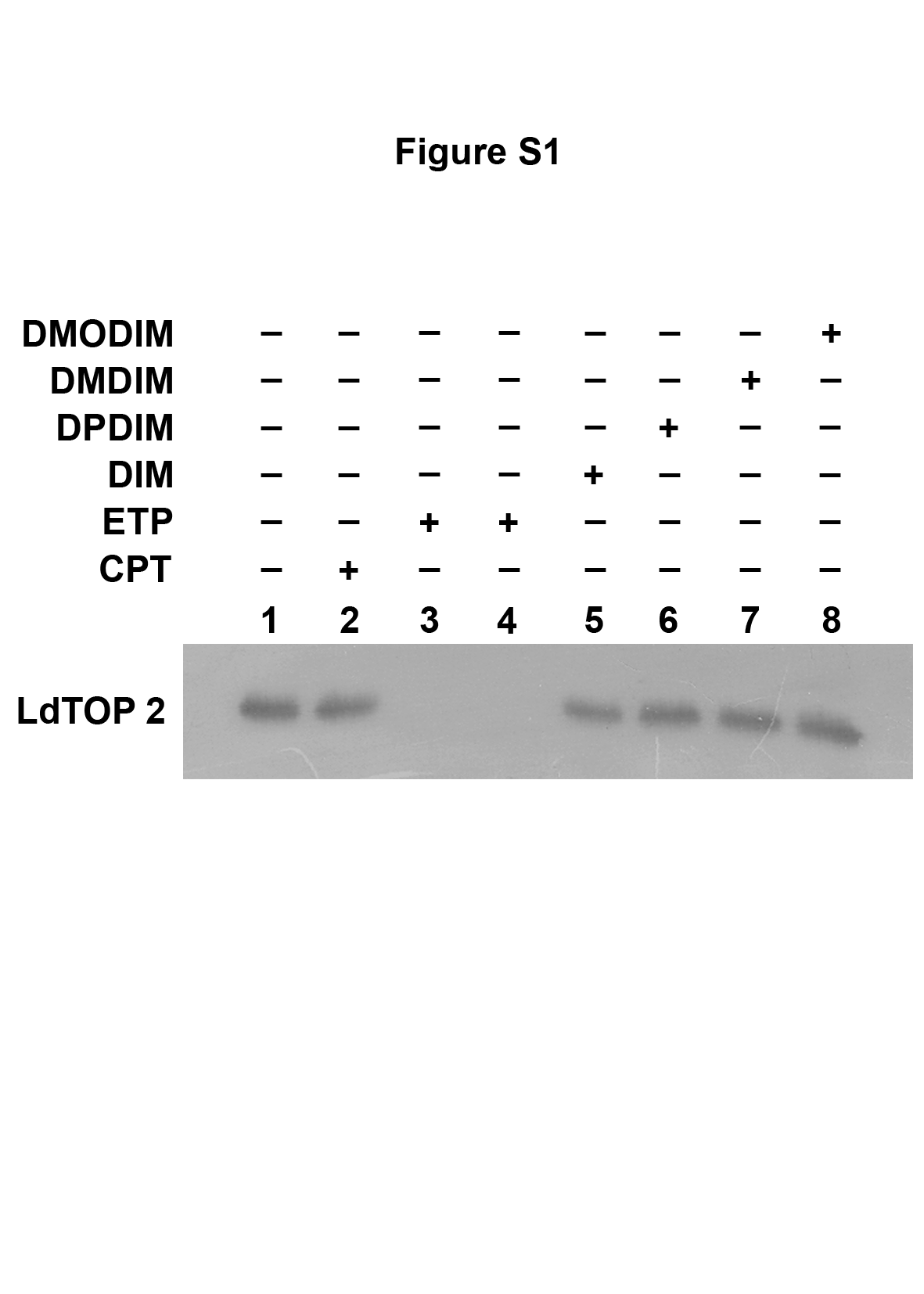

Supplement: Figure S1 — Stabilization of topoisomerase-II mediated cleavable complex was determined by immunoband depletion assay. Immunoband depletion of L. donovani topoisomerase II, using an antibody raised against LdTOP2 (ATPase domain, 43 kDa). Leishmanial cells were treated with 0.2% DMSO alone (lane 1), 20 µM CPT (lane 2); 20 µM and 50 µM of Etoposide (lanes 3 and 4 respectively) and 50 µM of DIM (lane 5), DPDIM (lane 6), DMDIM (lane 7) and DMODIM (lane 8) respectively. (TIF) [file pone.0028493.s001.tif]
